# Hemin Improves Insulin Sensitivity and Lipid Metabolism in Cultured Hepatocytes and Mice Fed a High-Fat Diet

**DOI:** 10.3390/nu9080805

**Published:** 2017-07-26

**Authors:** Yi Luan, Fang Zhang, Yalan Cheng, Jun Liu, Rui Huang, Menghong Yan, Yuangao Wang, Zhishui He, Hejin Lai, Hui Wang, Hao Ying, Feifan Guo, Qiwei Zhai

**Affiliations:** 1 Key Laboratory of Nutrition and Metabolism, CAS Center for Excellence in Molecular Cell Sciences, Institute for Nutritional Sciences, Shanghai Institutes for Biological Sciences, Chinese Academy of Sciences, University of Chinese Academy of Sciences, 320 Yueyang Road, Shanghai 200031, China; luanyi@sibs.ac.cn (Y.L.); fzhang@sibs.ac.cn (F.Z.); ylcheng01@sibs.ac.cn (Y.C.); liujunsinging@163.com (J.L.); huangrui@sibs.ac.cn (R.H.); mhyan@sibs.ac.cn (M.Y.); wangyuangao@sibs.ac.cn (Y.W.); zshe@sibs.ac.cn (Z.H.); hjlai@sibs.ac.cn (H.L.); hwang@sibs.ac.cn (H.W.); yinghao@sibs.ac.cn (H.Y.); ffguo@sibs.ac.cn (F.G.); 2 School of Life Science and Technology, Shanghai Tech University, Shanghai 200093, China

**Keywords:** hemin, insulin resistance, lipid metabolism, hepatic steatosis

## Abstract

Hemin is a breakdown product of hemoglobin. It has been reported that the injection of hemin improves lipid metabolism and insulin sensitivity in various genetic models. However, the effect of hemin supplementation in food on lipid metabolism and insulin sensitivity is still unclear, and whether hemin directly affects cellular insulin sensitivity is yet to be elucidated. Here we show that hemin enhances insulin-induced phosphorylation of insulin receptors, Akt, Gsk3β, FoxO1 and cytoplasmic translocation of FoxO1 in cultured primary hepatocytes under insulin-resistant conditions. Furthermore, hemin diminishes the accumulation of triglyceride and increases in free fatty acid content in primary hepatocytes induced by palmitate. Oral administration of hemin decreases body weight, energy intake, blood glucose and triglyceride levels, and improves insulin and glucose tolerance as well as hepatic insulin signaling and hepatic steatosis in male mice fed a high-fat diet. In addition, hemin treatment decreases the mRNA and protein levels of some hepatic genes involved in lipogenic regulation, fatty acid synthesis and storage, and increases the mRNA level and enzyme activity of CPT1 involved in fatty acid oxidation. These data demonstrate that hemin can improve lipid metabolism and insulin sensitivity in both cultured hepatocytes and mice fed a high-fat diet, and show the potential beneficial effects of hemin from food on lipid and glucose metabolism.

## 1. Introduction

Hemin is the oxidized form of heme, and is mainly synthesized in the mitochondria of blood cells and hepatocytes [1]. Heme is well-known as the iron-containing prosthetic group of hemoproteins such as hemoglobin [2]. Hemin is also a breakdown product of hemoglobin.

Hemin can selectively bind to some proteins such as transcription factors, kinases and receptors, and thereby alter gene transcription, protein synthesis and related biological functions [3]. Hemin binds to the transcriptional repressor Bach1, and regulates the transcription of some antioxidant enzymes [4]. Hemin interferes with the hemin-regulated inhibitor, a protein kinase that can specifically phosphorylate the initiation factor eIF2 and inhibit the initiation of new protein chains, and thus enhances protein synthesis [5]. Hemin binds to the nuclear hormone receptor Rev-erbα to downregulate genes involved in gluconeogenesis and fatty acid biosynthesis, and decreases glucose production in hepatoma cells [6,7]. Hemin binds to RXRα, and causes the impairment of RXRα-dependent signal pathways and inhibits the adipocyte differentiation of 3T3-L1 cells [8]. However, the effect of hemin on glucose and lipid metabolism still needs further investigation.

Recent evidence has highlighted the important role of hemin in glucose and lipid metabolism. Intraperitoneal injection of hemin for about 4 weeks lowered blood pressure, decreased glycemia and increased plasma insulin and insulin sensitivity in spontaneously hypertensive rat, a strain used as a model of essential hypertension, and hypertensive rats induced by uninephrectomy, deoxycorticosterone acetate and salt [9,10]. Furthermore, intraperitoneal injection of hemin for 4–5 weeks decreased glycemia and improved glucose and insulin tolerance in streptozotocin-induced diabetic rats, Zucker Diabetic Fatty rats, a model of type 2 diabetes with a mutation in leptin receptor and Goto-Kakizaki rats, a nonobese insulin resistant type 2 diabetic model [11,12,13]. Moreover, intraperitoneal injection of hemin at 30 mg/kg twice a week for 8 weeks reduces hyperleptinemia and leptin resistance in rats fed a high-fat diet (HFD) [14]. In addition, intraperitoneal injection of hemin at 50 μmol/kg daily for 4 weeks improves insulin sensitivity in skeletal muscle in mice fed HFD [15]. However, whether hemin has effect on hepatic insulin sensitivity and steatosis are yet to be elucidated and whether supplementation of hemin in food will has similar anti-diabetic effect as intraperitoneal injection is still unclear.

We hypothesized that oral administration of hemin would have beneficial effects on insulin sensitivity and lipid metabolism in mice fed HFD, and hemin could directly improve hepatic insulin sensitivity and lipid metabolism. To verify the hypothesis, we investigated the effect of hemin on insulin sensitivity and triglyceride accumulation in primary hepatocytes, and the effect of supplementation of hemin in food on insulin sensitivity and lipid metabolism in mice fed HFD. We show that hemin alleviates insulin resistance and diminishes triglyceride accumulation in primary hepatocytes induced by palmitate, and supplementation of hemin decreases body weight, energy intake, blood glucose, serum and liver triglyceride, and improves insulin and glucose tolerance and hepatic insulin signaling and lipid metabolism in mice fed HFD.

## 2. Materials and Methods

### 2.1. Cell Culture and Treatments

Mouse primary hepatocytes were prepared from male C57BL/6 at the age of 8–10 weeks as described previously with minor modifications [16]. Male mice were anesthetized with a single intraperitoneal injection of 50 mg/kg sodium pentobarbital, and the liver was perfused with Krebs-Ringer buffer with glucose (120 mM NaCl, 4.8 mM KCl, 1.2 mM MgSO_4_, 1.2 mM KH_2_PO_4_, 24 mM NaHCO_3_, 20 mM glucose, and 5 mM HEPES, pH 7.45) containing 100 μM ethylene glycol-bis(2-aminoethyl ether)-*N,N,N',N'*-tetraacetic acid (EGTA) prewarmed in a 37 °C water bath at a flow rate of 3 mL/min for 15 min. Then Krebs-Ringer buffer with glucose containing 5 mM CaCl_2_, 1.2% BSA and 140 U/mL Type I collagenase (Worthington, Lakewood, NJ, USA) prewarmed in a 37 °C water bath was used to perfuse the liver for 20 min. Livers were removed and cut into small pieces, gently agitated in Dulbecco's Modified Eagle Medium (DMEM) containing 25 mM glucose to release the hepatocytes, and then filtered through a 70 μm cell strainer (Thermo Fisher, Waltham, MA, USA) into a 50 mL centrifuge tube. The tube was centrifuged at 50× *g* for 2 min at 4 °C, and the cell pellet was washed with 25 mL DMEM containing 25 mM glucose 3 times, and then resuspended in about 4 mL DMEM containing 25 mM glucose and mixed with an equal volume of 90% percoll (Sigma, St. Louis, MO, USA) in phosphate-buffered saline (PBS) followed by centrifugation at 50× *g* for 10 min at 4 °C. The supernatant was removed, and the cell pellets were washed with DMEM containing 25 mM glucose. Then the cells were seeded at a density of 2 × 10^5^ cells/well with DMEM containing 10% fetal bovine serum (Gibco, Carlsbad, CA, USA) in 12-well plates precoated overnight with 20 μg/mL Collagen Type I (Merck Millipore, Darmstadt, Germany).

Hepatocytes were treated with hemin from Sigma with a purity of 97% at the indicated concentrations in DMEM with 5.5 mM glucose and containing 10% fetal bovine serum for 6 h, then palmitate and BSA were added to a final concentration of 0.6 mM and 1.2% respectively for 18 h to induce insulin resistance. Subsequently, the cells were stimulated with or without 100 nM insulin (Sigma, St. Louis, MO, USA) for 15 min and then harvested for immunoblotting, or stimulated with insulin for 10 min and subsequently used for immunofluorescence. Hemin was dissolved in dimethyl sulfoxide (DMSO) at the concentration of 3 mM as a stock solution. DMSO as the vehicle was added to each well at the final concentration of 0.1%.

### 2.2. Immunoblotting

Equal volume of cell and liver lysates at the same protein concentrations with a total amount of 15-30 μg protein were separated by sodium dodecyl sulfate polyacrylamide gel electrophoresis (SDS-PAGE), transferred to polyvinylidene fluoride (PVDF) membranes (Merck Millipore, Darmstadt, Germany), blocked and detected with antibodies against Tyr1131/1146-phosphorylated insulin receptor β (p-InsR), Ser473-phosphorylated Akt (p-Akt), Ser9-phosphorylated glycogen synthase kinase 3β (p-Gsk3β), Thr24-phosphorylated FoxO1 (p-FoxO1), insulin receptor β (InsR), Akt, Gsk3β, FoxO1, ATP-citrate lyase (ACL), stearoyl-Coenzyme A desaturase 1 (SCD1) (Cell Signaling Technology, Beverly, MA, USA); CD36 (Abcam, Cambridge, MA, USA); PPARγ, SREBP1 (Santa Cruz Biotechnology, Dallas, TX, USA); and Actin (Sigma, St. Louis, MO, USA). The immune complexes were detected using a horseradish peroxidase-conjugated secondary antibody and visualized with a chemiluminescence reagent (Thermo, Waltham, MA, USA). Each blot shown in the figures is representative of at least three experiments. Protein quantification was analyzed by ImageJ (http://rsb.info.nih.gov/ij/index.html), and normalized to the corresponding total proteins.

### 2.3. Immunofluorescence

Cells were fixed in 4% paraformaldehyde in PBS for 15 min, and then washed with PBS for about 5 min three times. Thereafter, the cells were permeabilized with PBS containing 0.1% Triton X-100 for about 15 min, and incubated in PBS containing 0.1% Triton X-100 and 5% BSA for 1 h. Subsequently, the cells were incubated with FoxO1 antibody (Cell Signaling Technology, Beverly, MA, USA) in PBS containing 0.1% Triton X-100 and 5% BSA for 1 h. After washed with PBS containing 0.1% Triton X-100 for 5 min three times, the cells were incubated with Alexa Fluor Cy3 goat anti-rabbit IgG (Jackson ImmunoResearch, Bar Harbor, ME, USA) in PBS containing 0.1% Triton X-100 and 5% BSA for 1 h. After washed with PBS containing 0.1% Triton X-100 for 5 min three times, DAPI (Sigma, St. Louis, MO, USA) at a concentration of 0.5 μg/mL in PBS containing 0.1% Triton X-100 was used to stain the nuclei. Immunofluorescence images were obtained with an Olympus IX51 (Olympus, Tokyo, Japan) fluorescence microscope. Quantitative analysis of FoxO1 translocation was performed as described previously with minor modifications [17]. Images were taken from three independent experiments, and three images were randomly taken from each experiment for each condition. At least seven cells were quantified from the upper-left corner of each image. The fluorescence intensity of a 1.5 × 1.5 μm square in nuclei and a same-size square at the left side of the nuclei in cytoplasm in the same cell was quantified by ImageJ.

### 2.4. Measurements of Triglyceride, Cholesterol, High-Density Lipoprotein Cholesterol, Low-Density Lipoprotein Cholesterol, Aspartate Transaminase and Alanine Transaminase

Hepatic triglyceride was extracted as previously described with minor modifications [16]. Briefly, 40–50 mg of liver tissues or 2 × 10^5^ cells were homogenized in 400 μL isopropanol containing four porcelain beads with a diameter of 2 mm using TissueLyser II (Qiagen, Hilden, Germany) at 30 Hz for 10 min, and then the sample was centrifuged at 6000× *g* for 10 min to collect supernatant for the following assays: triglyceride, cholesterol, high-density lipoprotein cholesterol, low-density lipoprotein cholesterol, aspartate transaminase and alanine transaminase, and were determined by enzymatic assay kits (Shensuo Unf Medical Diagnostics Articles Co., Shanghai, China).

### 2.5. Measurements of Free Fatty Acid

For cell or tissue samples, 10^6^ cells or 10 mg tissue samples were homogenized in 200 μL chloroform containing 1% Triton X-100 using TissueLyser II (Qiagen, Hilden, Germany). Then the lysates were centrifuged at 13,000× *g* for 10 min. The organic phase (lower phase) was collected, and dried in air for about 5 h until the trace chloroform was completely removed. Free fatty acids in the dried lipid samples were measured using a Free Fatty Acid Quantification kit (BioVision, Milpitas, CA, USA).

### 2.6. Animals

All animals were maintained and used in accordance with the guidelines of the Institutional Animal Care and Use Committee of the Institute for Nutritional Sciences (Protocol number 2014-AN-5, approved on 8 December 2014). The animals were housed in individual cages with free access to a regular diet and water in a room at 22 ± 1 °C on a 12 h light/dark cycle. Male C57BL/6 mice were purchased from Slac, and were randomly divided into three groups. The mice at the age of 19 weeks were randomly assigned to feed a diet of chow or a high-fat diet (HFD) with 60% kcal fat (Research Diets) without or with 0.08% or 0.4% hemin (Sigma, St. Louis, MO, USA). Body weight and food intake were measured after they were fed HFD with or without hemin. The HFD was ground and mixed with the powder of hemin at the indicated concentrations, and then was pelleted for the following studies. It has been reported that beef contains heme iron at the concentration of 26.4 mg/kg [18], which is equivalent to the concentration of heme at 307 mg/kg. If all heme in the beef was oxidized to hemin and beef was consumed at 1 kg/day per 60 kg of body weight, the dose of hemin for human consumption is 5.12 mg/day/kg, which equals to the dose of 62.9 mg/day/kg of body weight or about 0.04% in diet for mice. Thus, in this study, we choose the doses of 0.08% and 0.4% hemin in diet, which are about 2 and 10 times of the dose in human with consumption of beef at 1 kg/day per 60 kg bodyweight.

After fed an HFD with or without hemin for 7 or 8 weeks, the mice were fasted for 16 h and 4 h respectively to perform glucose and insulin tolerance tests. Briefly, the mice were injected with either 2 g/kg of glucose or 0.75 U/kg of human insulin (Novo Nordisk, Bagsvaerd, Denmark) into the peritoneal cavity, and blood glucose levels were measured from the tail vein at the indicated times after the injection of glucose or insulin using the FreeStyle blood glucose monitoring system (TheraSense, Alameda, CA, USA).

The mice fed HFD with or without hemin for 8 weeks were fasted overnight for 16 h, and then killed to collect blood samples or injected intraperitoneally with PBS or human insulin at a dose of 5 U/kg. Fifteen minutes later, the mice were killed, and the liver samples were collected for immunoblot to detect insulin signaling.

### 2.7. Histology

Liver tissues were excised and fixed with 4% paraformaldehyde in PBS for 2 days and then dehydrated in PBS containing 30% sucrose for at least 16 h. Following paraffin embedding, the tissue sections were stained with hematoxylin and eosin. For Oil Red O staining, liver samples were freshly fixed in 4% paraformaldehyde and dehydrated by sucrose gradient followed by an optimal cutting temperature compound-cryosection (10 μm), stained with 0.2% Oil Red O in 60% of isopropanol for 15 min, counterstained with haematoxylin, and then washed three times.

### 2.8. RNA Isolation and Quantitative PCR

RNA was extracted using TRIzol reagent (Invitrogen, Carlsbad, CA, USA). Then the RNA was reverse-transcribed using M-MLV Reverse Transcriptase (Promega, Madison, WI, USA) with random hexamer primers. Quantitative polymerase chain reaction (PCR) was performed using FastStart Universal SYBR Green Master (Roche, Basel, Swiss) on an ABI Prism 7900 Sequence Detection System. The primers GTGCCGCCTGGAGAAACCT and TGAAGTCGCAGGAGACAACC were used to detect mouse *Gapdh*; GGAGCCATGGATTGCACATT and GGCCCGGGAAGTCACTGT for mouse *SREBP1c*; TCCGTGATGGAAGACCACTCGCAT, and CAGCAACCATTGGGTCAGCTCTTG for mouse *Pparγ*; TCTGCAGATCGCGTGGAG and CTTGTCCCGGCATAGCAAC for mouse *ChREBPβ*; ATGGGCTGTGATCGGAACTG and GTCTTCCCAATAAGCATGTCTCC for mouse *Cd36*; TGAATCTCACGCGCCTACTATG and ATGACCCTGTTGCCTCCAAAC for mouse *Acc*; TGGATGCCACAGCTGACTAC and GGTTCAGCAAGGTCAGCTTC for mouse *Acl*; AAGTTGCCCGAGTCAGAGAA and CGTCGAACTTGGAGAGATCC for mouse *Fas*; GAAAAGCAGTTCAACGAGAACG and AGATGCCGACCACCAAAGATA for mouse *Elovl6*; CTGTACGGGATCATACTGGTTCCC and CAGCCGAGCCTTGTAAGTTCTGTG for mouse *Scd1*; GCCCTTCGTGGGAAGGTGCTGCTA and CCGTCTCGCCAGCCATCCTCTGTG for mouse *Gpat*; CTCCTGGAAGAAACGCCTTATT and CACCTTGCAGTAGTTGGAACC for mouse *Cpt1*; CCCAGAACTACGGCGCTTC and CCACGCTGCTATTCTTTCCTT for mouse *Echs1*; CAGGAAGTAAGATGCCTGGAAC and TGCAGCAGTACCAAGTTTAGTG for mouse *Acat*; TGCCAGAGCTGATTGACATTC and GGCATACCAGAAGGTGGTGAG for mouse *Acs*. The quantitative PCR data were normalized to *Gapdh*.

### 2.9. Enzyme Activity Measurements

Carnitine palmitoyltransferase-1 (CPT1) activity was measured by coupling to CoASH release and its reaction with 4,4′-dipyridyldisulfide as previously described [19]. In brief, liver mitochondria were isolated from mouse liver using a tissue mitochondria isolation kit (Beyotime, Nanjing, China). Briefly, 5 μL of the isolated liver mitochondria at the protein concentration of 5 μg/μL were incubated with 195 μL reaction buffer containing 25 mM palmitoyl-CoA, 2 mM L-carnitine, 125 μM 4,4′-dipyridyldisulfide, and 2 mM KH_2_PO_4_ at pH 7.5. The absorbance at 324 nm was monitored in a 96-well plate at 37 °C for 15 min.

The long chain acyl-CoA synthetase (ACS) activity was measured by coupling with pyruvate kinase and lactate dehydrogenase to monitor the formation of NAD [20]. Briefly, 2 μL of liver lysates at the protein concentration of 5 μg/μL were mixed with 198 μL of the reaction mixture containing 25 mM HEPES, 2 mM oleate, 1 mM phosphoenolpyruvate, 100 mM KCl, 5 mM MgCl_2_, 4 mM ATP, 1 mM CoA, 0.4 mM NADH, 2 U/mL myokinase, 2 U/mL pyruvate kinase and 2 U/mL lactate dehydrogenase at pH 7.4 in a 96-well plate. The absorbance at 340 nm was monitored at 37 °C for 15 min. The enzyme activities were normalized to the respective protein concentration.

### 2.10. Statistical Analysis

Data are expressed as mean ± standard deviation of at least three independent experiments. Statistical significance was assessed by the Student’s *t*-test. Differences were considered statistically significant at *p <* 0.05.

## 3. Results

### 3.1. Hemin Attenuates Palmitate-Induced Insulin Resistance in Mouse Primary Hepatocytes

**Figure 1:**
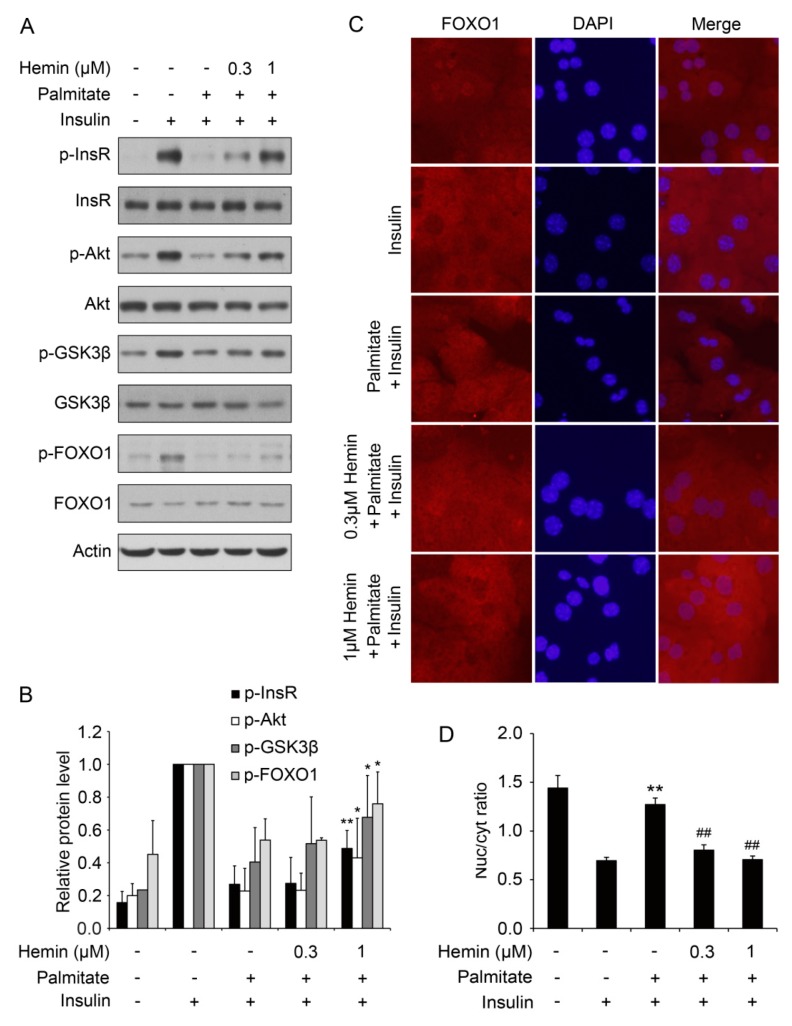
Hemin alleviates palmitate-induced insulin resistance in mouse primary hepatocytes. (**A**) Hemin improved insulin signaling, including the phosphorylation of InsR, Akt, Gsk3β and FoxO1 in hepatocytes under insulin-resistant conditions. Mouse primary hepatocytes were treated with hemin at the indicated concentration for 6 h, and then palmitate was added at a final concentration of 0.6 mM to induce insulin resistance for 18 h. Subsequently, the cells were stimulated with or without 100 nM insulin for 15 min and harvested for western blot; (**B**) Quantification of the protein level in (**A**). * *p* < 0.05; ** *p* < 0.01 versus hepatocytes stimulated by insulin with palmitate pretreatment and without hemin treatment; (**C**) Hemin enhances insulin-induced cytoplasmic translocation of FoxO1 under insulin resistance condition induced by palmitate. After treatment with hemin at the indicated concentration for 6 h, mouse primary hepatocytes were treated with 0.3 mM palmitate for 18 h, and then stimulated with 100 nM insulin for 10 min for immunofluorescence. Nuclei were stained with DAPI; (**D**) Quantitative analysis of FoxO1 translocation in (**C**). ** *p* < 0.01 versus hepatocytes stimulated with insulin without treatment of palmitate and hemin; ^##^ *p* < 0.01 versus hepatocytes stimulated by insulin with palmitate pretreatment and without hemin treatment. In this and all other figures, data are shown as mean ± standard deviation.

To investigate whether hemin can attenuate insulin resistance in cultured cells, we treated mouse primary hepatocytes with palmitate to induce insulin resistance as described previously [21]. As shown in Figure 1A,B, palmitate induced insulin resistance as indicated by impaired insulin-induced phosphorylation of insulin receptor, Akt, Gsk3β and FoxO1. Pretreatment with hemin at the concentration of 1 μM significantly attenuates palmitate-induced impaired insulin signaling (Figure 1A,B). These data show that hemin can attenuate insulin resistance induced by palmitate in hepatocytes.

To further confirm that hemin can attenuate insulin resistance in cultured cells, insulin-induced translocation of FoxO1 from nuclear to cytoplasm was detected by immunofluorescence. As shown in Figure 1C,D, palmitate markedly blocked insulin-induced translocation of FoxO1 from nuclear to cytoplasm. Pretreatment with hemin significantly reverse the effect of palmitate on translocation of FoxO1 (Figure 1C,D). These data further confirm that hemin can alleviate insulin resistance in cultured hepatocytes.

### 3.2. Hemin Inhibits Triglyceride Accumulation and Increase of Free Fatty Acid Content in Hepatocytes

**Figure 2:**
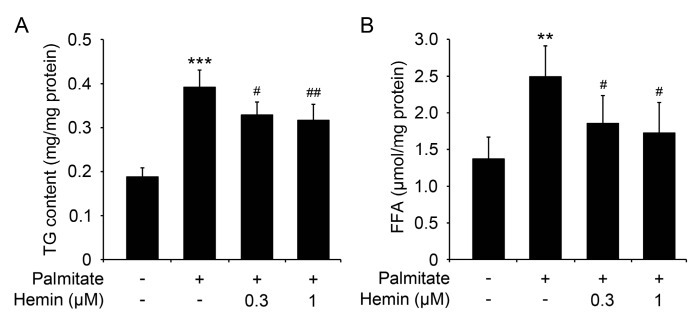
Hemin diminishes triglyceride accumulation and increases free fatty acid content in mouse primary hepatocytes induced by palmitate. Mouse primary hepatocytes were treated with hemin at the indicated concentration for 6 h, and then palmitate was added at a final concentration of 0.6 mM to induce the accumulation of triglyceride (TG) and increase of free fatty acid (FFA) for 18 h. Subsequently, the cells were harvested to measure cellular triglyceride (**A**) and free fatty acid (**B**). ** *p* < 0.01; *** *p* < 0.001 versus hepatocytes without treatment of palmitate and hemin; ^#^ *p* < 0.05; ^##^ *p* < 0.01 versus hepatocytes treated with palmitate but without hemin.

Hepatocytes treated with palmitate usually will lead to accumulation of triglyceride and increase of cellular free fatty acid level [16,22]. As hemin can attenuate palmitate-induced insulin resistance, we further investigated the effect of hemin on palmitate-induced accumulation of triglyceride and increase of cellular free fatty acid level. As shown in Figure 2A, palmitate markedly increased the cellular triglyceride level, and pretreatment with hemin significantly blocked the accumulation of triglyceride induced by palmitate. Moreover, palmitate markedly increased the cellular free fatty acid level, and pretreatment with hemin also significantly blocked the increase of free fatty acid induced by palmitate (Figure 2B). These data show that hemin can protect hepatocytes from palmitate-induced disorders of lipid metabolism.

### 3.3. Supplementation of Hemin Downregulates Body Weight, Energy Intake, Blood Glucose and Improves Insulin Sensitivity and Glucose Tolerance in Mice Fed a High-Fat Diet (HFD)

**Figure 3:**
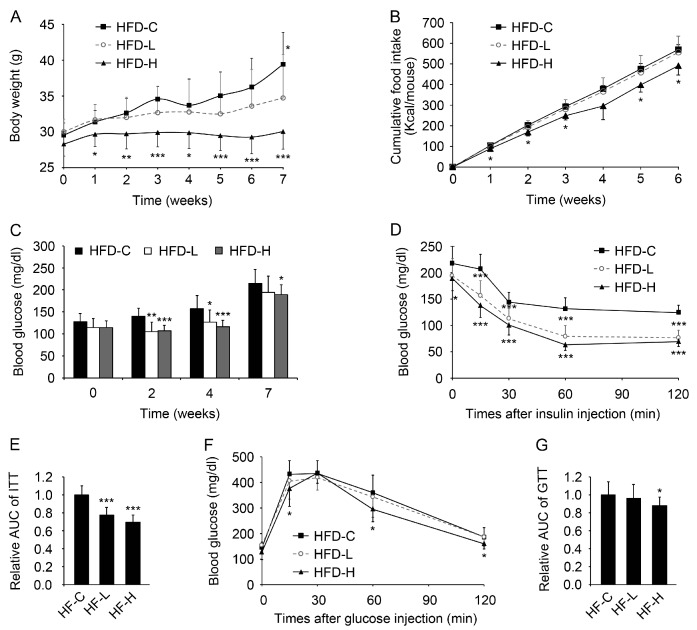
Hemin decreases body weight, energy intake, blood glucose and improves insulin and glucose tolerance in mice fed a high-fat diet (HFD). (**A**) The effect of hemin on body weight of mice fed HFD. Mice were fed HFD (HFD-C) or HFD supplemented with hemin at the low (HFD-L) or high dose (HFD-H); (**B**) The effect of hemin on total energy intake of mice fed HFD; (**C**) The effect of hemin on fasting blood glucose of mice fed HFD; (**D**,**E**) The effect of hemin on insulin tolerance of mice fed HFD for 7 weeks. ITT, insulin tolerance test; (**F**,**G**) The effect of hemin on glucose tolerance of mice fed HFD for 8 weeks. GTT, glucose tolerance test. * *p* < 0.05; ** *p* < 0.01; *** *p* < 0.001 versus HFD-C. *n* = 10 for each group in the above mouse studies.

To further investigate the effect of hemin on insulin sensitivity and glucose and lipid metabolism, mice were fed with HFD or HFD supplemented with hemin at the low or high dose as described in Materials and Methods [23]. As shown in Figure 3A,B, supplementation of hemin at a high dose markedly reduced body weight and energy intake. Supplementation of hemin at both low and high doses significantly alleviated high blood glucose induced by HFD (Figure 3C). As expected, supplementation of hemin at both low and high doses also dramatically improved insulin tolerance in mice fed HFD (Figure 3D,E). However, only supplementation of hemin at the high dose improved glucose tolerance in mice fed HFD (Figure 3F,G).

**Figure 4:**
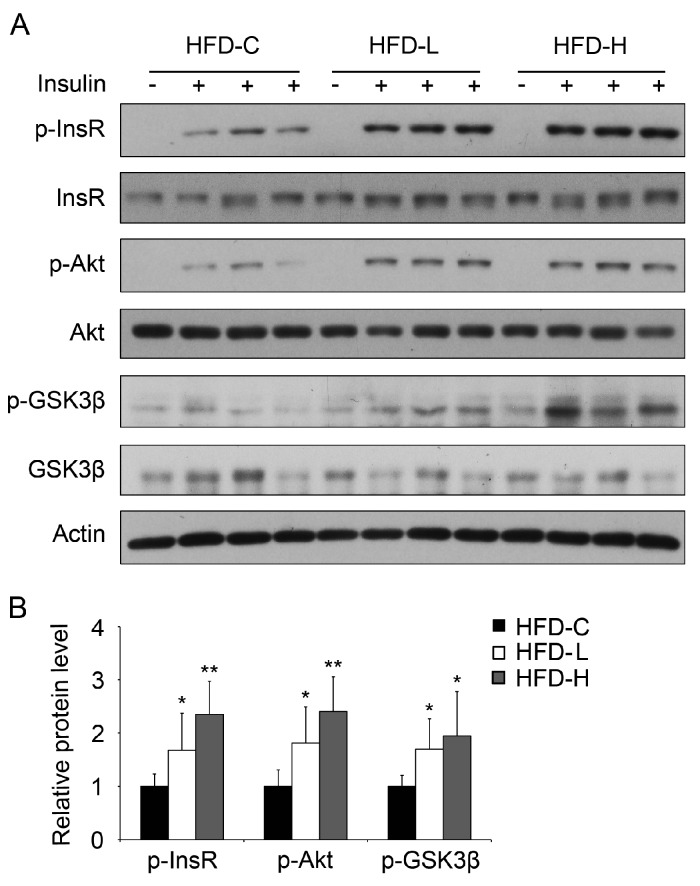
Hemin treatment improves insulin signaling in the liver of HFD mice. (**A**) Mice were fed HFD (HFD-C) or HFD supplemented with hemin at a low (HFD-L) or high dose (HFD-H) for 8 weeks. After fasting for 16 h, mice were injected with or without insulin for 15 min. Subsequently, liver samples were collected for western blot; (**B**) Quantification of p-InsR, p-Akt and p-Gsk3β protein levels in (**A**). * *p* < 0.05; ** *p* < 0.01. *n* = 6 for each group.

To further validate the effect of hemin on insulin sensitivity in vivo, we investigated the effect of supplementation of hemin on hepatic insulin signaling in mice fed HFD. As shown in Figure 4A,B, insulin-induced phosphorylation of insulin receptors Akt and Gsk3β were significantly upregulated in mice fed with HFD with supplementation of both low and high doses of hemin compared with the mice without hemin supplementation.

These data demonstrate that hemin supplementation can reverse HFD-induced increase of body weight and blood glucose, impaired glucose tolerance and insulin resistance.

### 3.4. Supplementation of Hemin Attenuates Hypertriglyceridemia and Hepatic Steatosis in Mice Fed HFD

**Figure 5:**
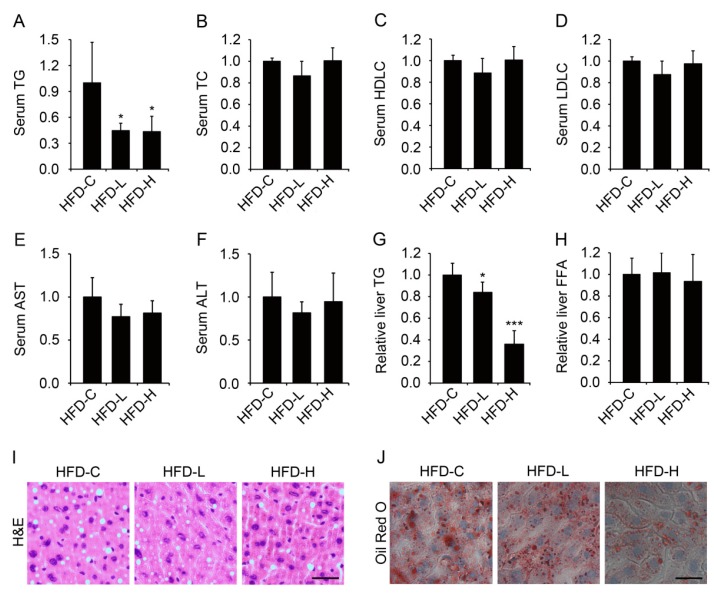
Hemin treatment downregulates serum triglyceride and attenuates hepatic steatosis in mice fed HFD. Mice were fed HFD (HFD-C) or HFD supplemented with hemin at a low (HFD-L) or high dose (HFD-H) for 8 weeks. Then, after fasted for 16 h, serum and liver samples were collected to measure serum triglyceride (TG) (**A**), serum total cholesterol (TC) (**B**), serum high-density lipoprotein cholesterol (HDLC) (**C**), serum low-density lipoprotein cholesterol (LDLC) (**D**), serum aspartate transaminase (AST) (**E**), alanine transaminase (ALT) (**F**), liver TG (**G**) and free fatty acid (**H**), or for hematoxylin and eosin staining (**I**) and oil red staining (**J**). Scale bar, 30 μm. * *p* < 0.05; *** *p* < 0.001 versus HFD-C. *n* = 4–6 for each group.

We next examined the effects of hemin on lipid metabolism in mice fed HFD. As shown in Figure 5A, the levels of serum triglyceride in mice fed HFD were significantly decreased with the supplementation of hemin, whereas supplementation of hemin had no significant effect on the serum levels of total cholesterol, high-density lipoprotein cholesterol, low-density lipoprotein cholesterol, aspartate transaminase and alanine transaminase in mice fed HFD (Figure 5B–F). Furthermore, we found that supplementation of hemin significantly reduced triglyceride content in liver of mice fed HFD, whereas had no significant effect on free fatty acid level in liver of mice fed HFD (Figure 5G,H). Hematoxylin and eosin and Oil Red O staining of liver sections showed that supplementation of hemin significantly suppressed the accumulation of lipid droplets in liver (Figure 5I,J). Taken together, these results show that supplementation of hemin can alleviate hypertriglyceridemia and hepatic steatosis in mice fed HFD.

### 3.5. Hemin Reduces Lipogenesis, Fatty Acid Synthesis and Lipid Storage and Increases Fatty Acid Oxidation in Liver of Mice Fed HFD

**Figure 6:**
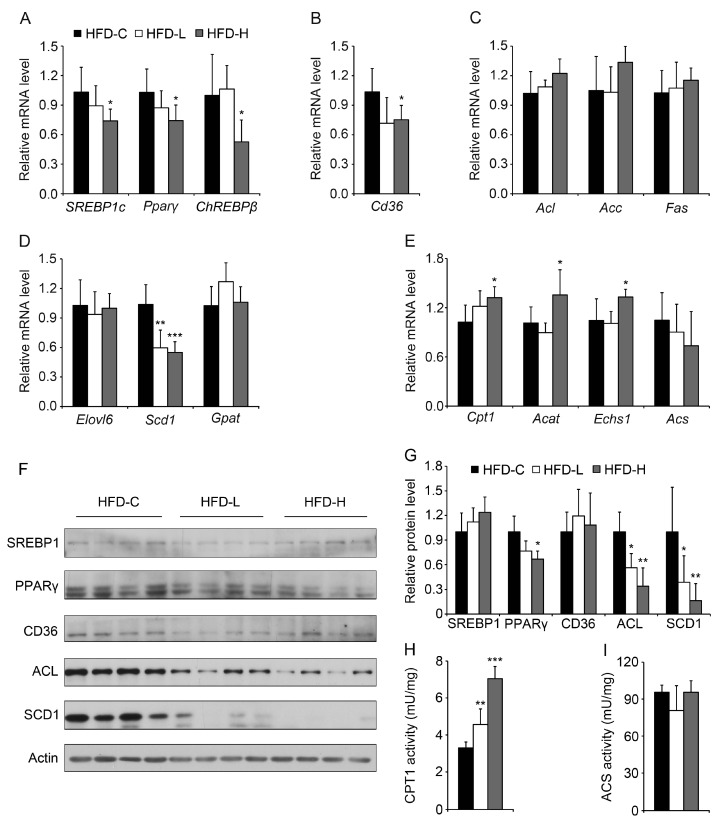
Hemin treatment decreases some hepatic genes involved in lipogenic regulation, fatty acid uptake and storage, and increases some hepatic genes involved in fatty acid oxidation. (**A**) Among the detected genes involved in lipogenic regulation, *SREBP1c*, *Pparγ* and *ChREBPβ* were downregulated by hemin in the liver of mice fed HFD as determined by quantitative PCR; (**B**) *Cd36* was downregulated by hemin in the liver of mice fed HFD; (**C**) The detected genes responsible for fatty acid synthesis, including *Acc*, *Acl* and *Fas*, showed no significant change; (**D**) As for the detected genes related to fatty acid storage, *Scd1* was markedly downregulated by hemin in the liver of mice fed HFD; (**E**) Among the detected genes involved in fatty acid oxidation, *Cpt1*, *Acat* and *Echs1* were upregulated by hemin in the liver of mice fed HFD. *n* = 6 for each group; (**F**,**G**) The protein levels of PPARγ, ACL and SCD1 were were significantly downregulated by hemin in the liver of mice fed HFD; (**H**,**I**) The enzyme activity of CPT1 (**H**), catalyzing the rate-limiting step in β-oxidation of fatty acids, but not that of ACS (**I**), was markedly upregulated by hemin in the liver of mice fed HFD. * *p* < 0.05; ** *p* < 0.01; *** *p* < 0.001.

To investigate the potential mechanisms of hemin on triglyceride accumulation in liver, first we examined the expression of some key regulators involved in lipogenesis. As shown in Figure 6A, both the lipogenic genes *SREBP1c*, *Pparγ* and *ChREBPβ* were significantly downregulated in liver of mice fed HFD with supplementation of high-dose hemin. Moreover, we found that *Cd36*, responsible for fatty acid uptake, decreased significantly in the liver of mice fed HFD with supplementation of high-dose hemin (Figure 6B). The mRNA levels of *Acl*, *Acc* and *Fas* responsible for fatty acid synthesis were unchanged in liver of mice fed HFD and hemin (Figure 6C), but the genes involved in fatty acid storage *Scd1*, but not *Elovl6* and *Gpat*, were downregulated in the liver of mice fed HFD with supplementation of hemin at both low and high doses (Figure 6D). In addition, the genes responsible for fatty acid oxidation, *Cpt1*, *Acat* and *Echs1*, but not *Acs* were upregulated in liver of mice fed HFD and high-dose hemin (Figure 6E). Then the protein levels of some genes involved in lipid metabolism were measured by immunoblot. The protein level of PPARγ was downregulated in the liver of mice fed HFD and high-concentration of hemin, but the hepatic protein levels of SREBP1 and CD36 were not significantly changed (Figure 6F,G). The protein levels of ACL and SCD1 were markedly decreased in the liver of mice fed HFD and hemin, suggesting the decrease of fatty acid synthesis and lipid storage. Consistently, CPT1 enzyme activity was significantly upregulated in the liver of mice fed HFD and hemin at both low and high doses (Figure 6H), whereas ACS enzyme activity was not significantly changed (Figure 6I). These data suggest that hemin reduces lipogenesis, fatty acid synthesis and lipid storage and increases fatty acid oxidation in liver of mice fed HFD, which might contribute to protective effect of hemin on liver steatosis in mice fed HFD.

## 4. Discussion

In the present study, we observed that hemin attenuated palmitate-induced insulin resistance, triglyceride accumulation and increase of free fatty acid content in mouse primary hepatocytes, and supplementation of hemin in food alleviated hyperglycemia, hypertriglyceridemia, insulin resistance and hepatic steatosis in mice fed HFD. These observations provide further insights for the effect of hemin on insulin sensitivity and lipid metabolism.

Previous studies have shown that intraperitoneal injection of hemin improves glucose and lipid metabolism in two models of hypertensive rats [9,10] and in type 1 diabetic rats and genetic obese and nonobese type-2 diabetic rats [11,12,13]. Here we show that supplementation of hemin in food improves glucose and lipid metabolism in mice fed HFD. Our data provide the evidence that oral administration of hemin can improve glucose and lipid metabolism in a diet-induced type 2 diabetes model, which implicated the potential beneficial effects of hemin from natural food or supplementation on glucose and lipid metabolism. Whether oral administration of hemin also has beneficial effects on glucose and lipid metabolism in other models of high fat feeding needs to be investigated in the future. It has been reported that hemin is an active ingredient of a biologic therapeutic approved by the Food and Drug Administration for the treatment of acute porphyries [24], which provides the possibility for the potential clinical application of hemin to treat diseases with disorders of glucose and lipid metabolism.

At cellular level, heme at 10 μM increases adipogenesis in human mesenchymal stem cells and mouse pre-adipocytes 3T3-L1 [25], and hemin at 60 μM also enhances the adipose differentiation of mouse pre-adipocytes 3T3-F442A [26]. Hemin at 25 μM binds to RXRα, and causes the impairment of RXRα-dependent signal pathways and inhibits the adipocyte differentiation of 3T3-L1 cells [8]. Hemin at 3 μM suppressed steatosis, reduced levels of alanine aminotransferase and lipid peroxidation induced by methionine- and choline- deficient medium in cultured hepatic cells [27]. Here, we found hemin at 0.3 and 1 μM alleviates palmitate-induced triglyceride accumulation and the increased intracellular free fatty acid level in cultured primary hepatocytes. The different effects of hemin on triglyceride accumulation might be due to the different cell type, culture condition and the concentration of hemin. In addition, we also found that supplementation of hemin significantly decreased body weight, serum and liver triglyceride levels in mice fed HFD, which is consistent with our observation at cellular level. Interestingly, we found that hemin is able to reduce the increase of FFA induced by palmitate in primary hepatocytes, while can not protect the hepatic accumulation of FFA in mice fed HFD. This discrepancy might be due to the different assay system performed in vitro and in vivo.

It has been reported that hemin can reverse the inhibitory effect of pretreatment with high glucose on glucose-stimulated insulin secretion in INS-1 cells [28]. Here, we found that hemin affects insulin signaling, and alleviates palmitate-induced insulin resistance in cultured primary hepatocytes and HFD-induced insulin resistance. In addition, hemin has been reported to induce the expression of heme oxygenase-1 and regulate insulin sensitivity and lipid metabolism [27,29,30], and whether heme oxygenase-1 also mediates the effects of hemin in our study needs to be confirmed in the future.

It has been reported that MALAT1 promotes hepatic steatosis and insulin resistance by increasing the stability of nuclear SREBP1-c, a key protein involved in fatty acid synthesis [31]. Hepatic overexpression of an active mutant form of CPT1A-enhanced hepatic fatty acid oxidation, which resulted in reduced liver triglyceride content and improved hepatic insulin signaling [32]. Similarly, we also observed the attenuated hepatic steatosis and the decrease of *SREBP1c*, *Pparγ*, *Scd1* and *Cd36* involved in lipogenesis and lipid uptake, and increase of *Cpt1*, *Acat* and *Echs1* involved in fatty acid oxidation in the liver of mice fed HFD and hemin. Meanwhile, the protein levels of ACL, PPARγ and SCD1 were downregulated in the livers of mice fed HFD and hemin. Consistently, CPT1 enzyme activity was also significantly upregulated in the livers of the mice fed HFD and hemin. The previous reports and our findings suggest that the inhibitory effect of hemin on intracellular triglyceride accumulation in palmitate-treated primary hepatocytes on the livers of mice fed HFD might contribute to the improved cellular and hepatic and whole body insulin sensitivity. Taken together, we found hemin can improve lipid metabolism and insulin sensitivity in cultured hepatocytes, and oral administration of hemin can improve lipid metabolism and insulin sensitivity in a diet-induced type 2 diabetes model. These findings provide the potential beneficial effects of hemin from food on lipid and glucose metabolism.
